# Laquinimod protects the optic nerve and retina in an experimental autoimmune encephalomyelitis model

**DOI:** 10.1186/s12974-018-1208-3

**Published:** 2018-06-14

**Authors:** Anna T. Wilmes, Sabrina Reinehr, Sandra Kühn, Xiomara Pedreiturria, Laura Petrikowski, Simon Faissner, Ilya Ayzenberg, Gesa Stute, Ralf Gold, H. Burkhard Dick, Ingo Kleiter, Stephanie C. Joachim

**Affiliations:** 1 Experimental Eye Research Institute, University Eye Hospital, Ruhr-University Bochum, In der Schornau 23-25, 44892 Bochum, Germany; 2 Department of Neurology, St. Josef-Hospital, Ruhr-University Bochum, Gudrunstrasse 56, 44791 Bochum, Germany

**Keywords:** Multiple sclerosis, Laquinimod, EAE, Optic nerve, Inflammation, Demyelination, Retinal degeneration, Glia response, Protection, Electroretinogram

## Abstract

**Background:**

The oral immunomodulatory agent laquinimod is currently evaluated for multiple sclerosis (MS) treatment. Phase II and III studies demonstrated a reduction of degenerative processes. In addition to anti-inflammatory effects, laquinimod might have neuroprotective properties, but its impact on the visual system, which is often affected by MS, is unknown. The aim of our study was to investigate potential protective effects of laquinimod on the optic nerve and retina in an experimental autoimmune encephalomyelitis (EAE) model.

**Methods:**

We induced EAE in C57/BL6 mice via MOG_35–55_ immunization. Animals were divided into an untreated EAE group, three EAE groups receiving laquinimod (1, 5, or 25 mg/kg daily), starting the day post-immunization, and a non-immunized control group. Thirty days post-immunization, scotopic electroretinograms were carried out, and mice were sacrificed for histopathology (HE, LFB), immunohistochemistry (MBP, Iba1, Tmem119, F4/80, GFAP, vimentin, Brn-3a, cleaved caspase 3) of the optic nerve and retina, and retinal qRT-PCR analyses (*Brn-3a*, *Iba1*, *Tmem119*, *AMWAP*, *CD68*, *GFAP*). To evaluate the effect of a therapeutic approach, EAE animals were treated with 25 mg/kg laquinimod from day 16 when 60% of the animals had developed clinical signs of EAE.

**Results:**

Laquinimod reduced neurological EAE symptoms and improved the neuronal electrical output of the inner nuclear layer compared to untreated EAE mice. Furthermore, cellular infiltration, especially recruited phagocytes, and demyelination in the optic nerve were reduced. Microglia were diminished in optic nerve and retina. Retinal macroglial signal was reduced under treatment, whereas in the optic nerve macroglia were not affected. Additionally, laquinimod preserved retinal ganglion cells and reduced apoptosis. A later treatment with laquinimod in a therapeutic approach led to a reduction of clinical signs and to an improved b-wave amplitude. However, no changes in cellular infiltration and demyelination of the optic nerves were observed. Also, the number of retinal ganglion cells remained unaltered.

**Conclusion:**

From our study, we deduce neuroprotective and anti-inflammatory effects of laquinimod on the optic nerve and retina in EAE mice, when animals were treated before any clinical signs were noted. Given the fact that the visual system is frequently affected by MS, the agent might be an interesting subject of further neuro-ophthalmic investigations.

**Electronic supplementary material:**

The online version of this article (10.1186/s12974-018-1208-3) contains supplementary material, which is available to authorized users.

## Background

Multiple sclerosis (MS) is a neurodegenerative and inflammatory disease of the central nervous system affecting more than 2.5 million people worldwide [1, 2]. The precise etiology of MS is not fully understood. Research of the past decades indicates a multifactorial background, comprising an impact of genetics, environmental factors, and gender [3].

The most important pathomechanism in MS is an autoimmune demyelination which is linked to inflammatory cell migration and the formation of central nervous system (CNS) white matter lesions [4]. Glial activation and lymphocytic infiltration play an important role in this process [5]. Clinically, MS patients display a large variety of symptoms [6]. As an evolutionary part of the CNS, the eye is also frequently involved. Optic neuritis, mainly unilateral, is the initial symptom in appr. 30% of MS patients and affects 60 to 70% in the later course [7]. It can manifest as subacute vision impairment up to complete loss of vision, central scotoma, diminished color vision, decreased contrast sensitivity, and retrobulbar pain during eye movement [5, 7, 8]. Papilledema and a relative afferent pupillary defect give diagnostic clues in ophthalmic examination [7, 9]. Recovery from optic neuritis is common, yet residual deficits can remain and impact quality of life [10, 11].

In MS research, experimental autoimmune encephalomyelitis (EAE) is the most common animal model. EAE is induced by immunization with CNS-specific antigens [12]. A murine EAE model, with myelin oligodendrocyte glycoprotein (MOG)_35–55_ as antigen, is known to induce chronic-progressive disease courses in C57BL/6 mice and affects spinal cord and optic nerves [13]. In the optic nerve, EAE is accompanied with demyelination and inflammatory cell infiltration [14–16]. Additionally, increased numbers of microglia in both optic nerve and retina [17], and a decrease of retinal ganglion cells [15] are common.

The oral drugs fingolimod, dimethyl fumarate, and teriflunomide have recently been introduced as MS therapies and are already broadly applied [18]. Another oral substance currently developed for MS is laquinimod, a quinoline-3-carboxamide [19]. Immunomodulatory, anti-inflammatory, and neuroprotective effects were observed in several EAE models [20–22]. In line with these results, phase II and phase III clinical trials demonstrated a reduction of active MRI lesions, less brain atrophy, and lower annualized relapse rates as well as less progression of disability in MS patients receiving laquinimod [23–25].

It is unknown whether inflammatory demyelination in the visual system, a crucial spot of manifestation in MS, is also affected by laquinimod therapy. This study aims at investigating therapeutic effects of laquinimod when applied at two different points in time on optic nerve and retina in a murine model of MS.

## Methods

### Animals

All experiments that involved animals were performed in compliance with the ARVO statement for the Use of Animals in Ophthalmic and Vision Research and approved by the animal care committee of North Rhine-Westphalia, Germany. C57BL/6 mice (Janvier, Paris, France) were housed in our facility under environmentally controlled conditions with free access to food and water ad libitum in the absence of pathogens.

### Induction and evaluation of EAE

To induce EAE, 10-weeks-old C57BL/6 mice (wild type) were immunized subcutaneously with 100 μg MOG_35–55_ peptide (provided by Charité, Berlin, Germany) in complete Freund’s adjuvant (BD Difco, Franklin Lakes, NJ, USA) containing 100 μg *mycobacterium tuberculosis H37Ra* (BD Difco). Additionally, mice received 500 ng pertussis toxin (Merck Millipore, Darmstadt, Germany) intraperitoneally on days 0 and 2 [26].

Immunized animals were divided into the following groups: one untreated EAE group and three EAE groups receiving laquinimod (Selleckchem, Munich, Germany) in doses of 1, 5, or 25 mg/kg body weight, respectively. Laquinimod was dissolved in 200 μl H_2_O and administered orally once per day, starting from the day after immunization. A non-immunized control group received PBS instead of MOG_35–55_ peptide and 200 μl H_2_O daily as a stress equivalent. 11–12 animals/group were analyzed. To investigate the effect of delayed treatment, animals were immunized with MOG_35–55_ peptide, as described above. When 60% of the animals had developed clinical signs of EAE (day 16), they were divided in two groups: EAE (*n* = 3) and Laq (*n* = 5). The animals in the Laq group received 25 mg/kg laquinimod.

Clinical assessment of EAE was performed daily, using a 10-point score system [27]: 0 = normal, 1 = less lively, 2 = impaired righting/limp tail, 3 = absent righting, 4 = ataxic gait, abnormal position, 5 = mild paraparesis, 6 = moderate paraparesis, 7 = severe paraplegia, 8 = tetraparesis, 9 = moribund, and 10 = death. Thirty days after MOG immunization, mice were sacrificed. For histology and immunohistochemistry, mice were perfused with 4% paraformaldehyde (Sigma-Aldrich, Munich, Germany), and the eyes and optic nerves were removed, post-fixed in 4% paraformaldehyde (Merck, Darmstadt, Germany), drained in 30% sucrose (VWR, Langenfeld, Germany), embedded in Tissue Tec (Thermo Scientific, Cheshire, UK) and frozen at − 80 °C. The retinas used for the qRT-PCR were isolated from the surrounding tissue and frozen at − 80 °C.

### Electroretinograms

For scotopic electroretinogram (ERG) measurements, we monitored retinal function using full-field flash electroretinography (HMsERG system; OcuScience LLC, Rolla, MO, USA) 30 days after immunization in both studies [28]. ERGs were recorded at 0.1, 0.3, 1, 3, 10, and 25 cd.s/m^2^. After amplification, digitalization, and averaging of signals, ERGView software (Version 4.380R; OcuScience LLC) was applied to evaluate a- and b-wave amplitudes.

### Histopathological staining and scoring of optic nerve

Longitudinal cryo-sections of optic nerves (4 μm, 1 nerve/animal) were stained with hematoxylin and eosin (HE; Merck) or luxol fast blue (LFB; RAL Diagnostics, Martillac Cedex, France) in both studies. Three images of each optic nerve section (anterior, medial, posterior) were taken with an Axio Imager M1 microscope (Zeiss, Oberkochen, Germany) at a ×400 magnification (six sections per animal).

After masking with a random number code via Ant Renamer software (http://antp.be/software/renamer), pictures were evaluated. The extent of inflammatory cell infiltration was measured using an established 4-point score on the HE-stained sections [15, 29]: 0 = no infiltration, 1 = mild infiltration, 2 = moderate infiltration, 3 = severe infiltration, and 4 = massive infiltration with formation of cellular conglomerates. The degree of demyelination in LFB-stained sections was assessed as 0 = no demyelination, 1 = moderate demyelination, and 2 = severe demyelination [15].

### Immunohistochemistry of optic nerve and retina

Immunohistochemistry of longitudinal sections of the optic nerve (4 μm, one nerve/animal) and retinal cross-sections (10 μm, one retina/animal) was conducted as previously described [17]. Primary and secondary antibodies are listed in Table 1. Six sections per animal were used for each staining. In the optic nerve, three photos and in the retina, four photos per section were taken using the Axio Imager M1 microscope (Zeiss) for myelin basic protein (MBP) and GFAP staining in the optic nerve and the ApoTome.2 microscope (Zeiss) for all other stainings with a ×400 magnification, respectively. Again, all images were masked with a random number code using Ant Renamer software and cut with a predefined window (Corel Paint Shop Pro, V13; Corel Corporation; Ottawa, Canada).

**Table 1:**
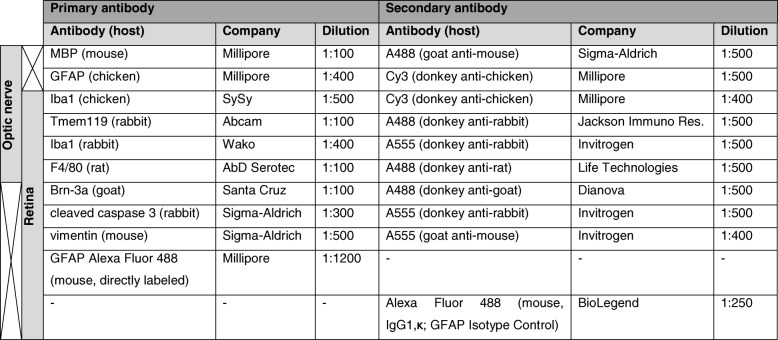
Antibodies used on optic nerve and retina for immunohistochemistry

Two different types of analyses were performed, both using ImageJ software (1.48v; Wayne Rasband National Institutes of Health, USA). With the first type, signal areas of MBP and GFAP in the optic nerve and of GFAP and vimentin in the retina were measured using an ImageJ macro [30, 31]. Briefly, the macro was set as follows: after transforming every photo into gray scale (32 bit), the level of background subtraction was averaged (MBP 14.47 pixels; GFAP optic nerve: 22.95 pixels, GFAP retina: 233.2 pixels; vimentin = 421 pixels) and the mean lower and upper threshold determined (MBP: lower threshold = 2.58, upper threshold = 33.33; GFAP optic nerve: lower threshold = 4.42, upper threshold = 60.91; GFAP retina: lower threshold = 10.08, upper threshold = 126.76; vimentin: lower threshold = 4.28, upper threshold = 93.51). Signals were measured as percentage of area. Regarding stainings of Brn-3a, cleaved caspase 3, Iba1, Tmem119, and F4/80, a second type of analysis was used: all cells labeled with the respective marker were counted with ImageJ cell counter (intern plugin of version 1.48v) in a masked fashion [32].

### Retinal quantitative real-time reverse transcription polymerase chain reaction

Both retinas of each animal were pooled for RNA preparation and cDNA synthesis as previously described [33]. The designed oligonucleotides for the quantitative real-time-PCR (qRT-PCR) are shown in Table 2. The qRT-PCR was performed using DyNAmo Flash SYBR Green (Thermo Scientific) on the PikoReal qRT-PCR Cycler (Thermo Scientific).

**Table 2:** Primer pairs for qRT-PCR analysis

Oligonucleotides	Sequence 5′ to 3′
*ß-actin*_fwd	ctaaggccaaccgtgaaaag
*ß-actin*_rev	accagaggcatacagggaca
*Brn-3a*_fwd	ctccctgagcacaagtaccc
*Brn-3a*_rev	ctggcgaagaggttgctc
*CD68*_fwd	tgatcttgctaggaccgctta
*CD68*_rev	taacggcctttttgtgagga
*Cyclophilin*_fwd	ttcttcataaccacagtcaagacc
*Cyclophilin*_rev	tccaccttccgtaccacatc
*Iba1*_fwd	ggatttgcagggaggaaaa
*Iba1*_rev	tgggatcatcgaggaattg
*GFAP*_fwd	acagactttctccaacctccag
*GFAP*_rev	ccttctgacacggatttggt
*Tmem119*_fwd	gtgtctaacaggccccagaa
*Tmem119*_rev	agccacgtggtatcaaggag
*AMWAP*_fwd	tttgatcactgtggggatga
*AMWAP*_rev	acactttctggtgaaggcttg

The primer pairs listed in the table were used in qRT-PCR experiments. *ß-actin* and *cyclophilin* served as housekeeping genes for retinal samples. *Fwd* forward, *rev* reverse

### Statistical analyses

Statistical analyses were carried out using Statistica software (V13; DELL, Tulsa, OK, USA) for ERGs and immunohistochemistry: groups were compared to each other by one-way ANOVA, followed by post hoc Tukey HSD test. HE and LFB score statistics comprised Kruskal-Wallis test followed by Dunn’s test using Graph Pad Prism 5 (San Diego, CA, USA). For qRT-PCR, statistical evaluation of threshold cycle (Ct) variations, and calculated relative expression variations, groups were analyzed by a pairwise fixed reallocation and randomization test using REST© software (Qiagen, Hilden, Germany) [34]. In the therapeutic treatment paradigm, EAE, LFB, and HE scores were evaluated using a non-parametric Mann-Whitney *U* test (Statistica) and ERGs and immunohistochemistry were compared using Student’s *t* test (Statistica). *P* values < 0.05 were considered as statistically significant. Data are presented as mean ± standard deviation (SD) for EAE scores, ERGs and immunohistochemistry and as median, interquartile range and range for qRT-PCR, and HE and LFB scores. Data of the second were presented as mean ± SD ± standard error (SEM) for ERG, HE and LFB scores and immunohistochemistry and as mean ± SD for EAE scores.

## Results

### Fewer neurological symptoms in mice receiving laquinimod

**Fig. 1:**
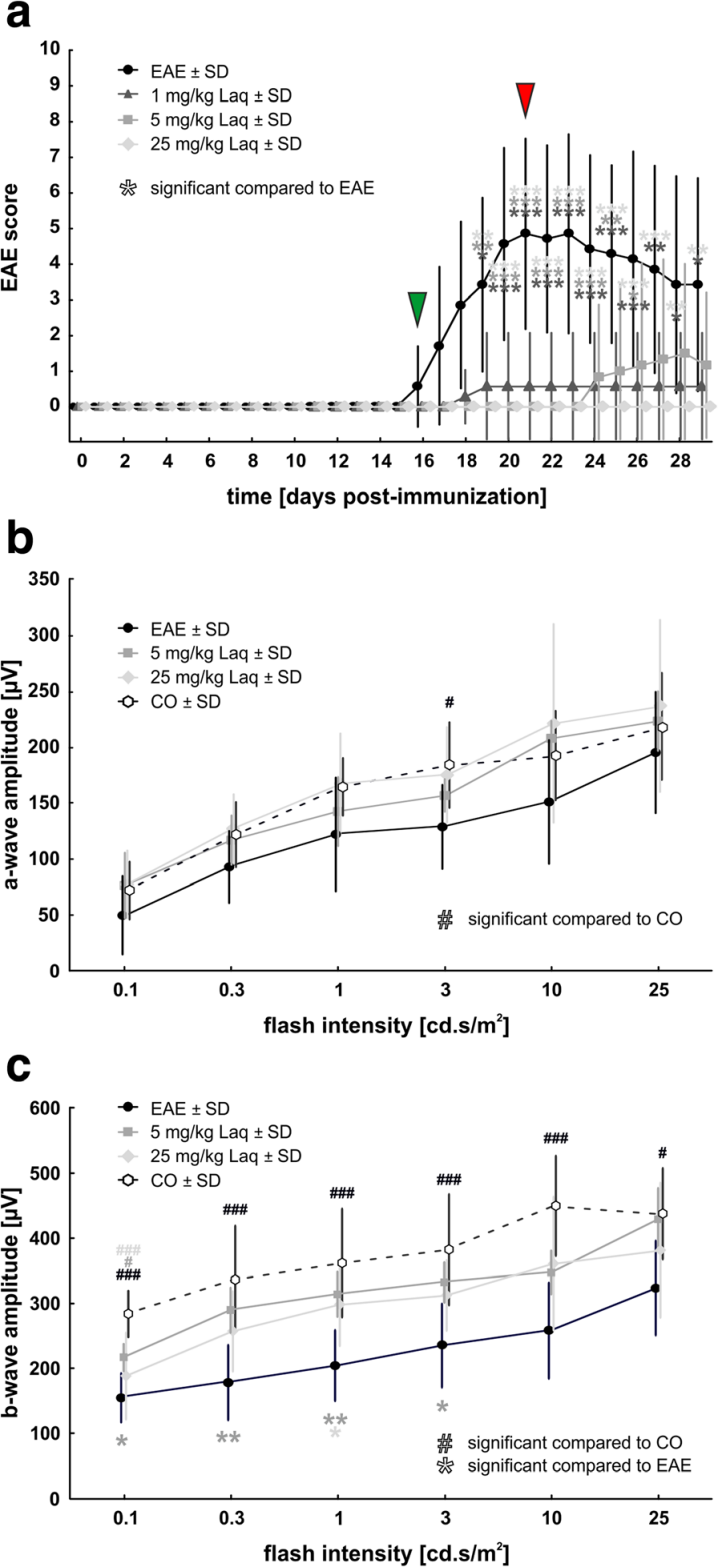
Clinical effects of laquinimod treatment. **a** Mean clinical EAE scores after immunization with MOG_35–55_ peptide. Green arrowhead: onset of symptoms. Red arrowhead: symptoms’ peak. **b** Electroretinograms were measured at the end of the experiment at day 30. A-wave amplitudes illustrate conductivity of photoreceptors. **c** B-wave amplitudes illustrate conductivity of the inner nuclear layer. Values represent mean ± SD. One-way ANOVA and Tukey post hoc. *N* = 6–7/group in **a** and *n* = 5/group in **b**, **c**. Comparison to control group: ^#^*p* < 0.05, ^###^*p* < 0.001. Comparison to untreated EAE group: *p < 0.05, **p < 0.01, ***p < 0.001

Mice developed clinical signs of EAE starting at day 16 after MOG_35–55_ immunization (Fig. 1a). The average score of EAE mice increased up to a plateau phase with its peak at days 21–23 (mean score day 21: 4.9 ± 2.7), equivalent with an ataxic gait and mild paraparesis of hind limbs. From day 25 on, a partial remission of disease was observed. In contrast to EAE mice, mice treated with laquinimod showed less neurological deficits. Their highest EAE scores measured 0.6 ± 1.5 for the 1 mg/kg laquinimod group (days 19–29), 1.5 ± 2.5 for the 5 mg/kg laquinimod group (day 28), and 0.0 ± 0.0 for the 25 mg/kg laquinimod group (all days). From days 19 to 29, EAE scores of treated groups were significantly lower than those of the EAE group with the most significant difference in the EAE plateau phase (days 20 to 24; *p* < 0.001 for all treated groups).

### Better electrical output of the inner nuclear layer in laquinimod-treated mice

A-wave and b-wave amplitudes were evaluated via ERG recording. The a-wave amplitude (Fig. 1b) represents the electrical output of photoreceptors. In the untreated EAE group, the a-wave amplitude was significantly reduced at 3 cd s/m^2^ flash intensity compared to the control group. Laquinimod-treated groups showed a slight but non-significant trend for improved electrical conductivity at all flash intensities compared to the untreated EAE group. No other effects on a-wave courses were measured. However, there were significant differences regarding the b-wave amplitude (Fig. 1c), which mirrors the electrical output of neurons of the inner nuclear layer. In the EAE group, the b-wave amplitude was strongly reduced at all flash intensities compared to the control group. Application of 5 mg/kg laquinimod constantly improved the electrical output in comparison to the untreated EAE group: the b-wave amplitude was significantly increased at flash intensities of 0.1 up to 3 cd s/m^2^ and an increasing trend was seen for the 10 and 25 cd s/m^2^ flashes. The 25 mg/kg laquinimod group displayed a significantly improved b-wave amplitude compared to the untreated EAE group at a flash intensity of 1 cd s/m^2^.

### Less cellular infiltration in the optic nerve with highly dosed laquinimod

**Fig. 2:**
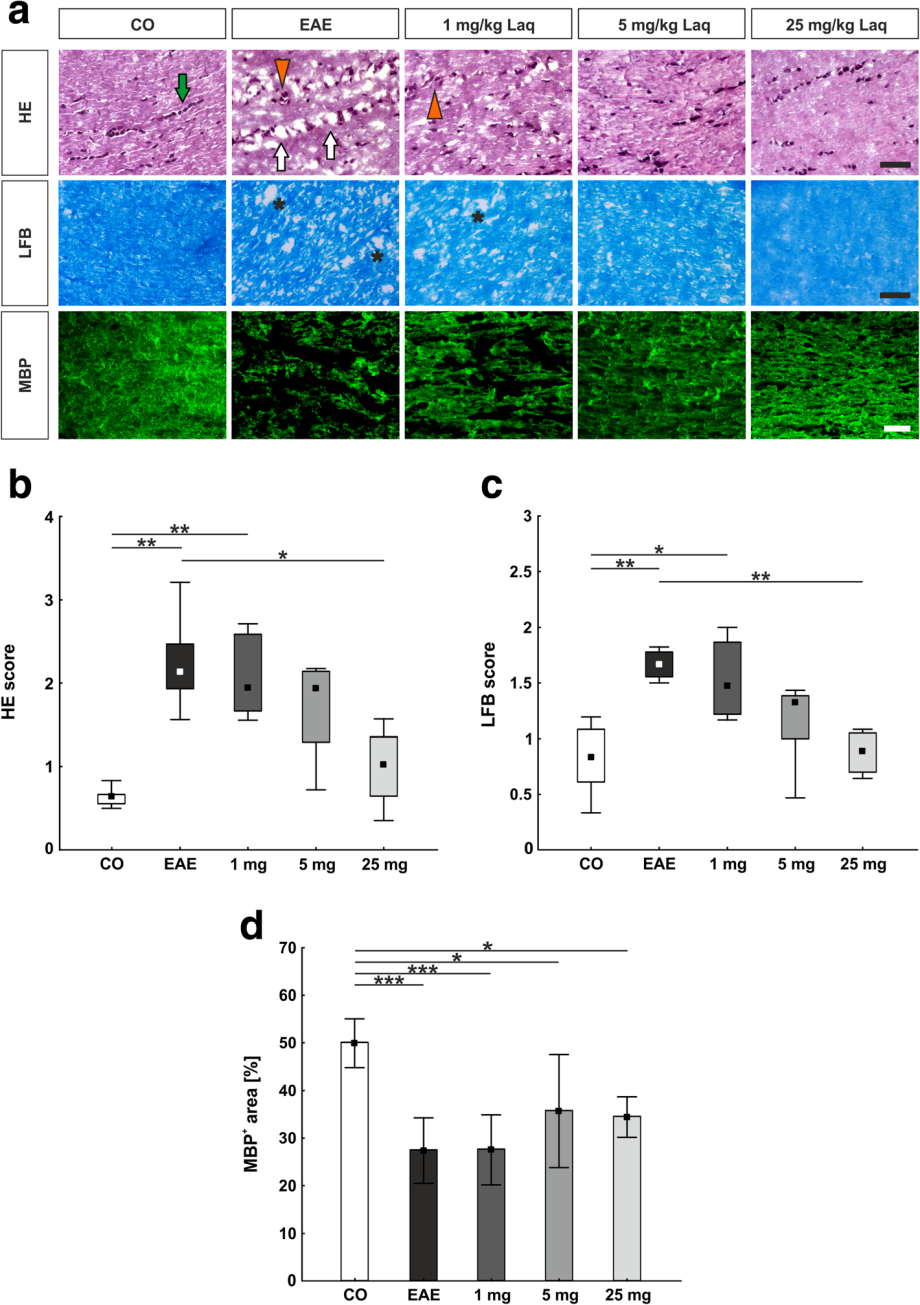
Structural preservation of the optic nerve in laquinimod-treated mice. **a** HE-stained healthy optic nerves show a linear formation of nuclei in a bead-like manner (green arrow), which in EAE is disrupted (white arrows) and might comprise cellular conglomerates (orange arrowheads). The usual structure of LFB-stained myelin sheaths in optic nerve tissue resembles combed bundles in a steady parallel arrangement (control group). In EAE, brightening in structure represents an interruption of this arrangement (star). Labeling of MBP, a main component of myelin sheaths, shows specific changes in myelin structure. **b** Cellular infiltration measured via HE score. **c** Demyelination measured via LFB score. **d** Demyelination measured via MBP staining. Values represent median, interquartile range, range in **b** and **c**, and mean ± SD in **d**. Kruskal-Wallis plus Dunn’s test for **b** and **c** and one-way ANOVA plus Tukey post hoc for **d**. *N* = 6–7/group. *p < 0.05, **p < 0.01, ***p < 0.001. Scale bars: 40 μm in HE and LFB, 20 μm in MBP

Sections of optic nerve tissue were stained with HE (Fig. 2a) and scored to evaluate the degree of cellular infiltration. The EAE group showed significantly more cellular infiltration, with a median score of 2.1 (interquartile range (IQR) 1.9–2.5) compared to the control group with a score of 0.6 (IQR 0.6–0.7; *p* < 0.01) (Fig. 2b). Application of 25 mg/kg laquinimod significantly decreased the cellular infiltration score to 1.0 (IQR 0.6–1.4; *p* < 0.05) in comparison to the EAE group. Smaller doses of laquinimod did not reduce cellular infiltration (1 mg/kg: 1.9, IQR 1.7–2.6; 5 mg/kg: 1.9, IQR 1.3–2.1; both *p* > 0.05 versus untreated EAE mice).

### Less demyelination in the optic nerve with highly dosed laquinimod

Histopathological investigations on optic nerve tissue also included LFB staining (Fig. 2a) followed by scoring to analyze the extent of demyelination. In the EAE group, a higher demyelination score of 1.7 (IQR 1.6–1.8) was found compared to the control group score of 0.8 (IQR 0.6–1.1; *p* < 0.01; Fig. 2c). Administration of laquinimod in the dose 25 mg/kg significantly diminished the degree of demyelination compared to the EAE group (0.9, IQR 0.7–1.1; *p* < 0.01), whereas medication with 1 and 5 mg/kg laquinimod revealed no significant reduction of demyelination (1 mg/kg: 1.5, IQR 1.2–1.9; 5 mg/kg: 1.3, IQR 1.0–1.4, both *p* > 0.05).

Myelin sheaths were also analyzed via myelin basic protein (MBP) labeling (Fig. 2a). The control group showed significantly more MBP signal (50.0 ± 5.1%/image), meaning preserved myelin, than all other groups (EAE: 27.5 ± 6.9%/image, *p* < 0.001; 1 mg/kg: 27.6 ± 7.4%/image, *p* < 0.001; 5 mg/kg: 35.8 ± 11.9%/image, *p* = 0.022; 25 mg/kg: 34.5 ± 4.3%/image, *p* = 0.011; Fig. 2d).

### Less microglia and recruited phagocytes in the optic nerve under laquinimod treatment

**Fig. 3:**
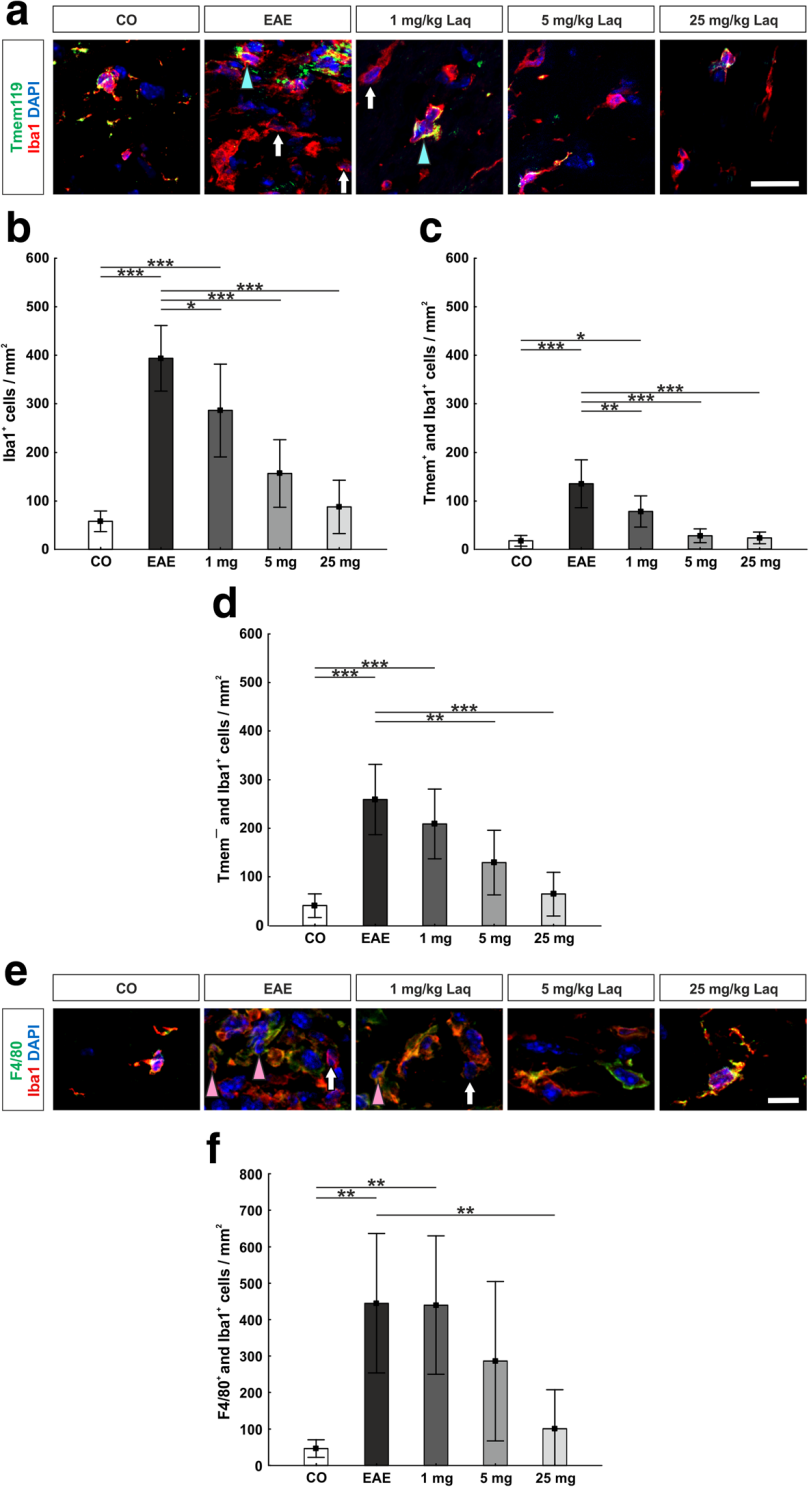
Less microglia and recruited phagocytes in the optic nerve under laquinimod treatment. **a** Iba1 antibody was used to label all phagocytes and combined with Tmem119 antibody to distinguish between microglia (Tmem^+^ and Iba1^+^, turquoise arrowheads) and recruited phagocytes (Tmem^−^ and Iba1^+^, white arrows). **b** Numbers of phagocytes/mm^2^. **c** Numbers of microglia/mm^2^. **d** Numbers of infiltrating phagocytes/mm^2^. All groups showed more infiltrating phagocytes than microglia. **e** Iba1 and F4/80 antibody to select cells with macrophage function (F4/80^+^ and Iba1^+^, pink arrowheads). **f** Numbers of cells with macrophage function/mm^2^. Values represent mean ± SD. One-way ANOVA and Tukey post hoc. *N* = 6–7/group. *p < 0.05, **p < 0.01, ***p < 0.001. Scale bars: 20 μm in **a**, 10 μm in **e**

All phagocytes were labeled with an Iba1 antibody. The co-staining with Tmem119 antibody was used to differentiate between resident microglia and recruited phagocytes [35] (Fig. 3a).

In the optic nerve, EAE animals presented significantly more Iba1^+^ cells than the control group (393.2 ± 67.7 cells/mm^2^ versus 57.8 ± 21.2 cells/mm^2^_;_ *p* < 0.001) (Fig. 3b). Administration of laquinimod significantly reduced their number compared to EAE animals (1 mg/kg: 285.8 ± 95.4 cells/mm^2^, *p* = 0.045; 5 mg/kg: 156.4 ± 69.6 cells/mm^2^, *p* < 0.001; 25 mg/kg: 87.4 ± 55.0 cells/mm^2^, *p* < 0.001).

Microglia (Tmem^+^ and Iba1^+^, Fig. 3c) formed a smaller part of all Iba1^+^ cells than infiltrating phagocytes (Tmem^−^ and Iba1^+^, Fig. 3d). In the EAE group, significantly more microglia (Tmem^+^ and Iba1^+^) were detected than in the control group (134.7 ± 49.4 cells/mm^2^ versus 17.0 ± 11.0 cells/mm^2^; *p* < 0.001). All laquinimod-treated groups showed significantly less Tmem^+^ and Iba1^+^ cells compared to EAE mice (1 mg/kg: 77.5 ± 32.2 cells/mm^2^, *p* = 0.009; 5 mg/kg: 27.4 ± 14.2 cells/mm^2^, *p* < 0.001; 25 mg/kg: 23.0 ± 12.1 cells/mm^2^, *p* < 0.001).

Similar results were observed for recruited phagocytes: the EAE group had significantly more Tmem^−^ and Iba1^+^ cells than the control group (258.5 ± 72.2 cells/mm^2^ versus 40.8 ± 23.9 cells/mm^2^; *p* < 0.001). Treatment with 5 and 25 mg/kg laquinimod significantly reduced the numbers of Tmem^−^ and Iba1^+^ cells compared to EAE (5 mg/kg: 128.9 ± 66.3 cells/mm^2^, *p* = 0.007; 25 mg/kg: 64.5 ± 44.5 cells/mm^2^, *p* < 0.001). The 1 mg/kg laquinimod group showed no significant effect (208.3 ± 71.6 cells/mm^2^, *p* > 0.05).

The co-staining of Iba1 with an F4/80 antibody was used to select cells with macrophage function (Fig. 3e). The EAE group expressed significantly more F4/80^+^ and Iba1^+^ cells than the control group (444.6 ± 191.2 cells/mm^2^ versus 46.4 ± 24.1 cells/mm^2^; *p* = 0.002) (Fig. 3f). Under application of 25 mg/kg laquinimod, significantly diminished numbers of F4/80^+^ and Iba1^+^ cells (100.8 ± 106.4 cells/mm^2^, *p* < 0.01) were detected compared to EAE mice, whereas lower doses showed no significant changes (1 mg/kg: 439.5 ± 189.8 cells/mm^2^; 5 mg/kg: 286.0 ± 218.6 cells/mm^2^; both *p* > 0.05).

### Laquinimod did not affect macroglia in the optic nerve

**Fig. 4:**
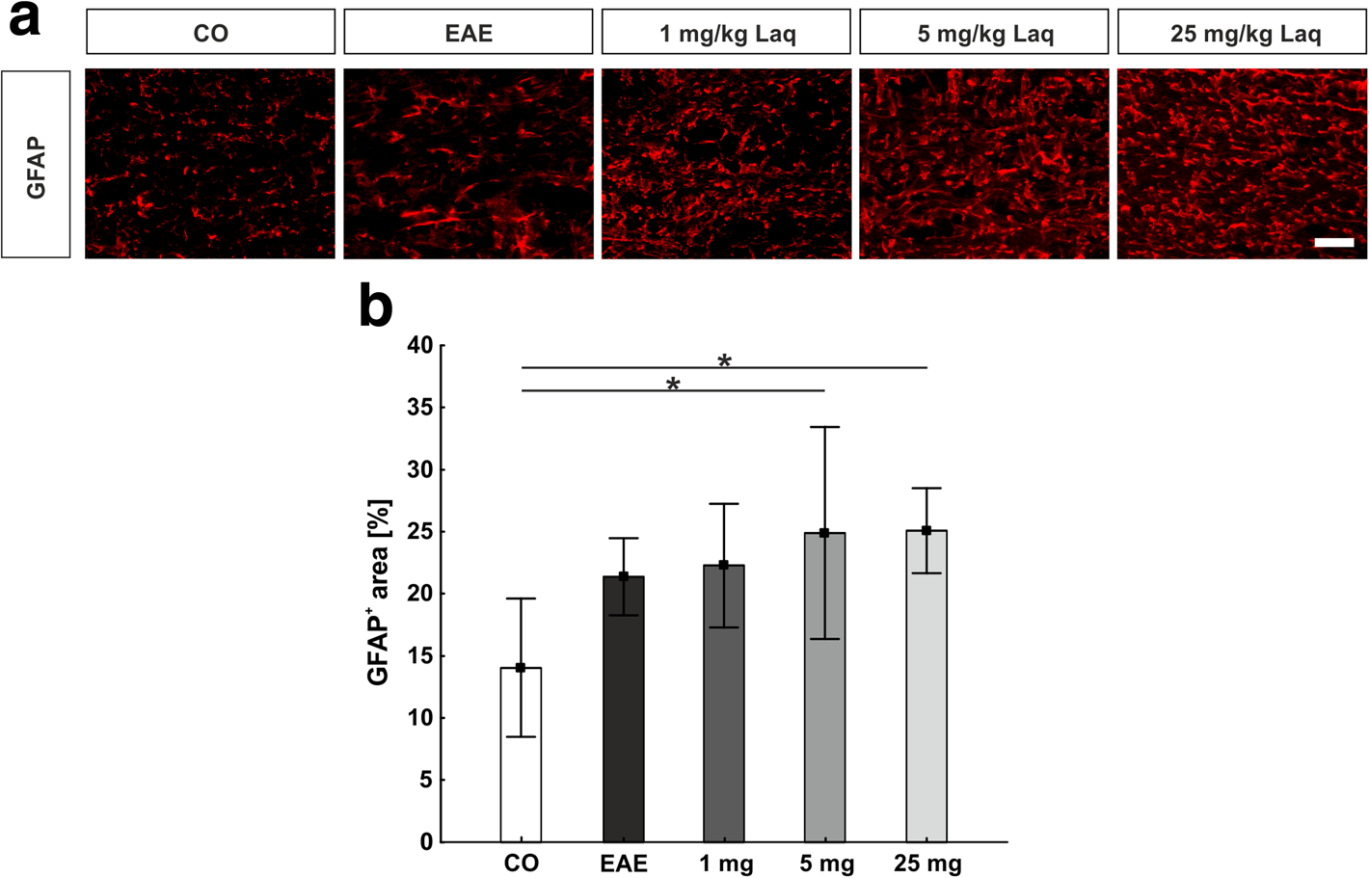
No change of macroglia in the optic nerve under laquinimod treatment. **a** Macroglia were labeled with GFAP antibody. **b** Macroglia signal area as percentage. Values represent mean ± SD. One-way ANOVA plus Tukey post hoc. *N* = 6–7/group. *p < 0.05. Scale bar: 20 μm

Macroglial cells were detected by labeling with a GFAP antibody (Fig. 4a). No significant changes in the GFAP^+^ area were found in mice treated with laquinimod compared to the EAE group (all *p* > 0.05; EAE: 21.4 ± 3.1%/image; 1 mg/kg: 22.3 ± 5.0%/image; 5 mg/kg: 24.9 ± 8.5%/image; 25 mg/kg: 25.1 ± 3.4%/image, control group: 14.0 ± 5.6%/image; Fig. 4b).

### Laquinimod reduced apoptosis and loss of retinal ganglion cells

**Fig. 5:**
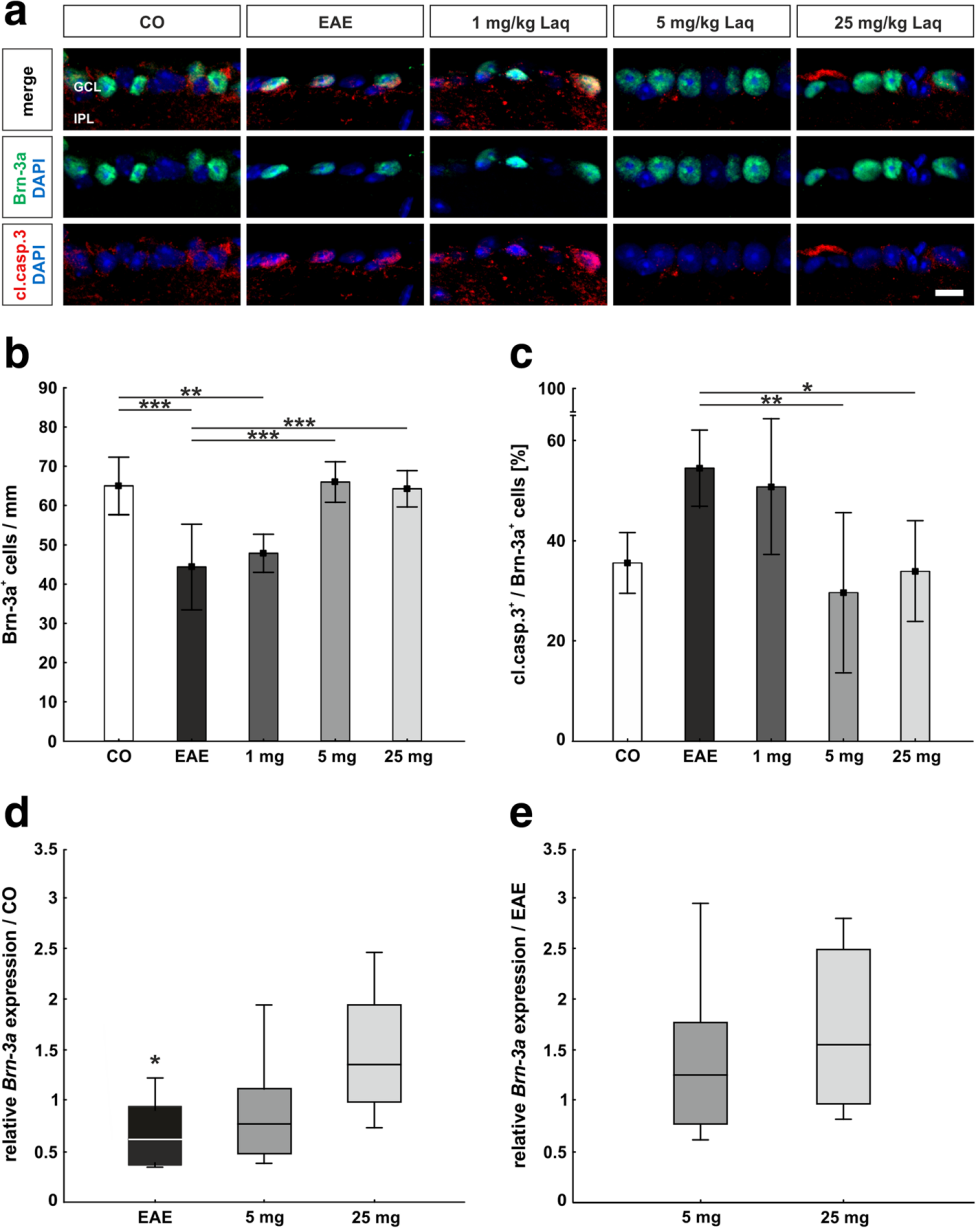
Protection of retinal ganglion cells under laquinimod treatment. **a** Retinal ganglion cells were marked with Brn-3a antibody (green), apoptotic cells via cleaved caspase 3 antibody (red). **b** Numbers of retinal ganglion cells/mm. **c** Percentage of apoptotic retinal ganglion cells. **d** *Brn-3a* expression compared to the control group. **e** *Brn-3a* expression compared to the EAE group. Values represent mean ± SD in **b** and **c** and median, interquartile range, range in **d** and **e**. One-way ANOVA plus Tukey post hoc for **b** and **c** and pairwise fixed reallocation and randomization test for **d** and **e**. *N* = 6–7/group in **a**–**c** and *n* = 5/group in **d** and **e**. *p < 0.01, **p < 0.01, ***p < 0.001. Scale bar: 10 μm. GCL = ganglion cell layer, IPL = inner plexiform layer

We used a Brn-3a antibody to mark retinal ganglion cells. Cleaved caspase 3 antibody was utilized to label apoptotic cells, and apoptotic retinal ganglion cells were positive for both markers (Fig. 5a). Additionally, *Brn-3a* mRNA expression was quantified via qRT-PCR. Compared to the control group, significantly less retinal ganglion cells were detected in EAE animals (64.9 ± 7.3 cells/mm versus 44.3 ± 10.9 cells/mm, *p* < 0.001; Fig. 5b). Under administration of 5 mg/kg laquinimod (65.9 ± 5.2 cells/mm, *p* < 0.001) and 25 mg/kg laquinimod (64.2 ± 4.6 cells/mm, *p* < 0.001), significantly higher numbers of retinal ganglion cells could be observed compared to the EAE group. For the group receiving 1 mg/kg laquinimod, no significant difference could be shown (47.8 ± 4.9 cells/mm, *p* = 0.886). In line with the results of Brn-3a immunostaining, qRT-PCR analyses displayed a significantly lower *Brn-3a* mRNA expression in EAE animals (0.62-fold expression) compared to the control group (*p* = 0.036; Fig. 5d; Additional file 1). Under the dose of 25 mg/kg laquinimod, the trend of an increased *Brn-3a* mRNA expression compared to the EAE group was noted (1.56-fold, *p* = 0.074; Fig. 5e; Additional file 1).

The percentage of apoptotic retinal ganglion cells from all retinal ganglion cells displayed a non-significant trend of higher fractions in the EAE group (54.4 ± 7.6%) compared to the control group (35.5 ± 6.1%, *p* = 0.051; Fig. 5c). Administration of laquinimod in doses of 5 (29.5 ± 16.0%, *p* = 0.006) and 25 mg/kg (33.9 ± 10.1%, *p* = 0.029) led to a significant reduction in the percentage of apoptotic retinal ganglion cells compared to EAE animals. Again, application of 1 mg/kg laquinimod showed no significant effect (50.8 ± 13.5%, *p* = 0.972).

### Less retinal microglia and macrophages under laquinimod treatment

**Fig. 6:**
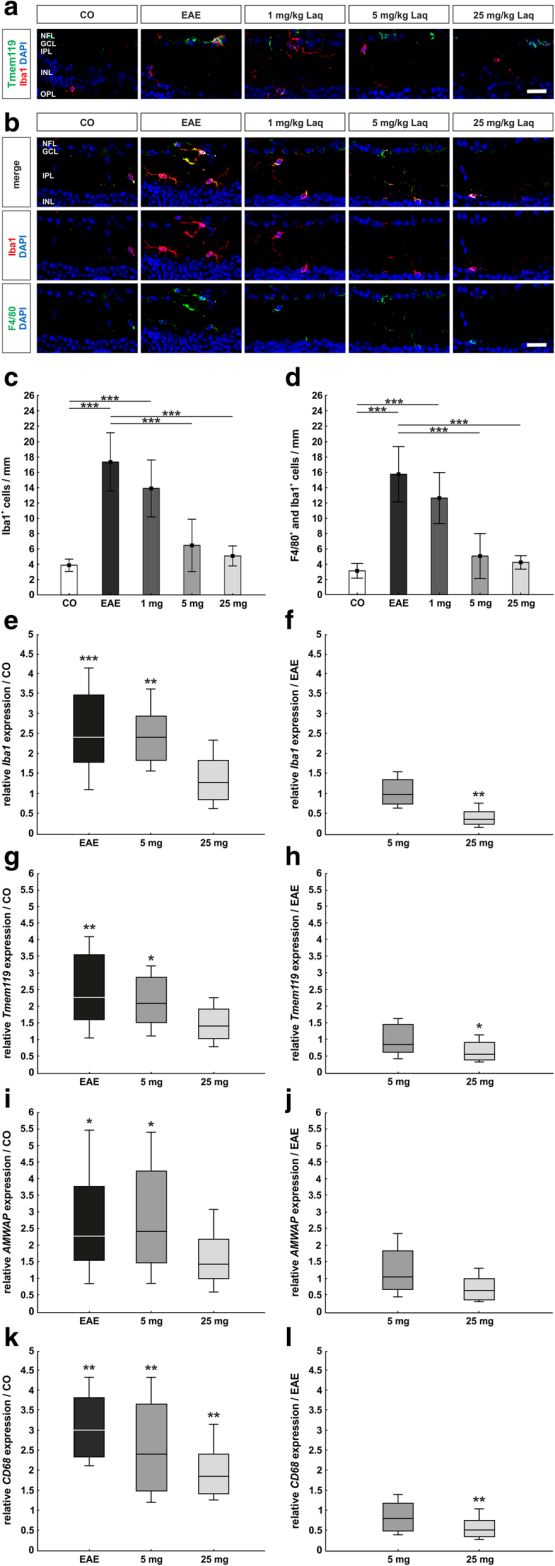
Less retinal microglia and macrophages under laquinimod treatment. **a** Exemplary staining of retinal Tmem119 (microglia) and Iba1 (phagocytes). **b** Iba 1 antibody (red) and F4/80 antibody (green) were co-stained to select cells with macrophage function. **c** Numbers of phagocytes/mm. **d** Numbers of cells with macrophage function/mm. **e** *Iba1* expression compared to the control group. **f** *Iba1* expression compared to the EAE group. **g** *Tmem119* expression compared to control. **h** *Tmem119* expression compared to EAE. **i** Expression of active microglia marker *AMWAP* compared to the control group. **j** *AMWAP* expression compared to EAE. **k** Macrophage marker *CD68* expression compared to the control group. **l** *CD68* expression compared to EAE. Values represent mean ± SD in **c** and **d** and median, interquartile range, range in **e**–**l**. One-way ANOVA plus Tukey post hoc for **c** and **d**; pairwise fixed reallocation and randomization test for **e**–**l**. *N* = 6–7/group in **b**–**d** and *n* = 5/group in **e**–**l**. *p < 0.01, **p < 0.01, ***p < 0.001. Scale bars: 20 μm. NFL = nerve fiber layer, GCL = ganglion cell layer, IPL = inner plexiform layer, INL = inner nuclear layer, OPL = outer plexiform layer

Similar to the optic nerve, we focused on retinal microglia via Tmem119 (overview staining, Fig. 6a). Retinal phagocytes were detected via Iba1 (Fig. 6a, b) and co-stained with the macrophage marker anti-F4/80 (Fig. 6b).

In EAE animals, significantly more Iba1^+^ phagocytes were counted than in the control group (17.3 ± 3.8 cells/mm versus 3.9 ± 0.8 cells/mm; *p* < 0.001) (Fig. 6c). Laquinimod in doses of 5 mg/kg (6.5 ± 3.4 cells/mm, *p* < 0.001) and 25 mg/kg (5.1 ± 1.3 cells/mm, *p* < 0.001) significantly reduced the number of phagocytes compared to EAE animals. The lowest laquinimod dose had no detectable impact (1 mg/kg: 13.9 ± 3.7 cells/mm, *p* = 0.223).

Co-staining of F4/80 and Iba1 revealed significantly more F4/80^+^ and Iba1^+^ cells in the EAE group than in the control group (15.7 ± 3.6 cells/mm versus 3.1 ± 1.0 cells/mm; *p* < 0.001; Fig. 6d). Under application of 5 and 25 mg laquinimod/kg, significantly diminished numbers of macrophages were detected than in the EAE group (5 mg/kg: 5.0 ± 2.9 cells/mm, 25 mg/kg: 4.2 ± 0.9 cells/mm; both *p* < 0.001). The lowest dose showed no significant difference (1 mg/kg: 12.6 ± 3.3 cells/mm, *p* = 0.227).

Moreover, retinal *Iba1*, *Tmem119* (microglia), *AMWAP* (activated microglia), and *CD68* (macrophages) mRNA expression was analyzed via qRT-PCR (Fig. 6e-l, Additional file 1). *Iba1* mRNA expression analysis corroborated the results of the Iba1-immunostaining. Not only the EAE group, but also animals receiving 5 mg/kg laquinimod showed significantly higher expressions of *Iba1* mRNA than control animals (EAE: 2.41-fold, *p* < 0.001; 5 mg/kg: 2.42-fold, *p* = 0.002; Fig. 6e; Additional file 1). A significantly lower *Iba1* mRNA expression than in EAE mice was only observed under application of 25 mg/kg laquinimod (0.38-fold, *p* = 0.003; Fig. 6f; Additional file 1).

Expression analyses of the microglia marker *Tmem119* showed a significantly higher expression of *Tmem119* mRNA in the EAE group and the 5 mg/kg laquinimod group compared to the control group (EAE: 2.27-fold, *p* = 0.006; 5 mg/kg: 2.11-fold, *p* = 0.01; Fig. 6; Additional file 1). Application of 25 mg/kg laquinimod significantly reduced *Tmem119* mRNA expression compared to EAE mice (0.57-fold, *p* = 0.038; Fig. 6h; Additional file 1).

The expression of *AMWAP*, a marker for active microglia [36], which rises in different models of retinal pathologies [37, 38] was increased in both EAE and 5 mg/kg laquinimod animals (EAE: 2.27-fold, *p* = 0.024; 5 mg/kg: 2.45-fold, *p* = 0.017; Fig. 6i; Additional file 1). Under therapy with 25 mg/kg laquinimod, a trend of diminished *AMWAP* mRNA expression compared to EAE mice was seen (0.64-fold, *p* = 0.06; Fig. 6j; Additional file 1).

In qRT-PCR expression analyses of the macrophage marker *CD68*, a significantly higher expression of *CD68* mRNA compared to the control group was not only found in the EAE group (3.01-fold, *p* = 0.002; Fig. 6k; Additional file 1), but also in the groups receiving 5 mg/kg laquinimod (2.41-fold, *p* = 0.002) and 25 mg/kg laquinimod (1.85-fold, *p* = 0.003). Animals treated with 25 mg/kg laquinimod expressed significantly less retinal *CD68* mRNA than the EAE group (0.53-fold, *p* = 0.008; Fig. 6l; Additional file 1).

### Diminished retinal macroglia response under application of laquinimod

**Fig. 7:**
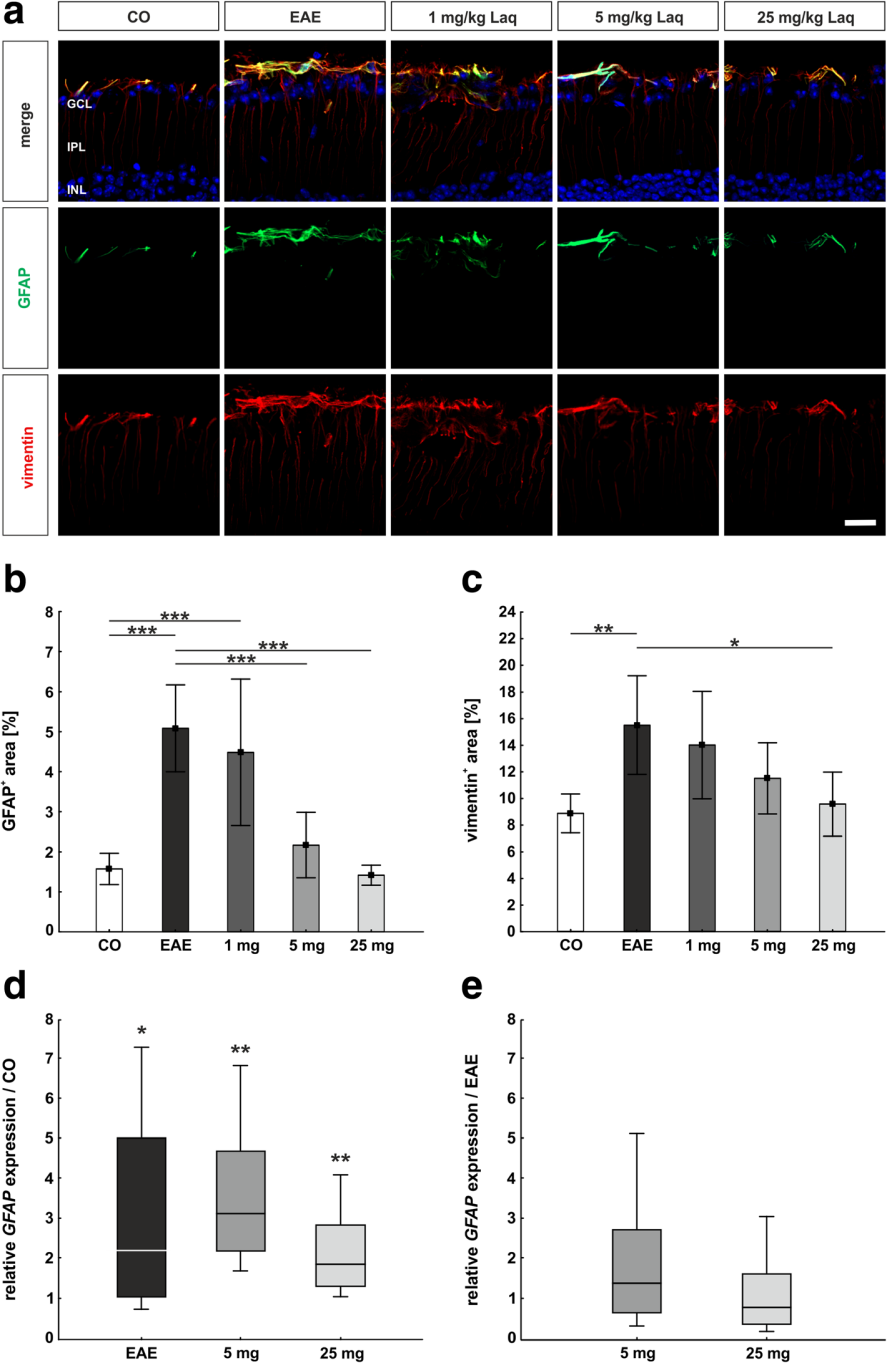
Less retinal macroglia response under laquinimod treatment. **a** GFAP antibody was applied to mark retinal macroglia (green). Retinal Müller glia was investigated via vimentin antibody (red). **b** Macroglia signal area as percentage. **c** Müller glia signal area as percentage. **d** *GFAP* expression compared to the control group. **e** *GFAP* expression compared to EAE. Values represent mean ± SD in **b** and **c** and median, interquartile range, range in **d** and **e**. One-way ANOVA plus Tukey post hoc for **b** and **c**; pairwise fixed reallocation and randomization test for **d** and **e**. *N* = 6–7/group in **a**–**c** and *n* = 5/group in **d** and **e**. *p < 0.05, **p < 0.01, ***p < 0.001. Scale bars: 20 μm. GCL = ganglion cell layer, IPL = inner plexiform layer, INL = inner nuclear layer

Retinal macroglia were detected via GFAP and vimentin (Fig. 7a). Moreover, retinal *GFAP* mRNA expression was analyzed via qRT-PCR (Additional file 1).

The EAE group showed a significantly larger GFAP^+^ macroglial signal area than the control group (5.1 ± 1.1%/image versus 1.6 ± 0.4%/image; *p* < 0.001; Fig. 7b). In the groups receiving 5 mg/kg laquinimod (2.2 ± 0.8%/image, *p* < 0.001) and 25 mg/kg laquinimod (1.4 ± 0.3%/image, *p* < 0.001), significantly less GFAP^+^ signal area was observed than in the EAE group. The group receiving 1 mg/kg laquinimod showed no significant difference compared to the EAE group (4.5 ± 1.8%/image, *p* = 0.833). The qRT-PCR analysis revealed significantly more *GFAP* mRNA in the EAE group (2.23-fold, *p* = 0.03; Fig. 7d; Additional file 1) compared to the control group. Also in the groups receiving laquinimod, a higher *GFAP* mRNA expression than in the control group was detected (5 mg/kg: 3.13-fold, *p* = 0.003; 25 mg/kg: 1.88-fold, *p* = 0.006; Fig. 7d; Additional file 1). Compared to the EAE group, no significant changes in *GFAP* mRNA expression were noted for treated mice (5 mg/kg: 1.40-fold, *p* = 0.4; 25 mg/kg: 0.84-fold, *p* = 0.6; Fig. 7e; Additional file 1).

We used a vimentin antibody to examine Müller glia in retinal tissue (Fig. 7a). The EAE group showed significantly more vimentin^+^ Müller glia signal area than the control group (15.5 ± 3.7%/image versus 8.9 ± 1.5%/image; *p* = 0.007) (Fig. 7c). Application of laquinimod in the dose of 25 mg/kg significantly decreased the area of vimentin signal (9.6 ± 2.4%/image, *p* = 0.019) in comparison to the EAE group. Smaller doses of laquinimod caused slight but non-significant reduction of Müller glia signal (1 mg: 14.0 ± 4.0%/image; 5 mg: 11.5 ± 2.7%/image; both *p* > 0.05).

### Later therapeutic treatment led to a decreased EAE score and preservation of retinal function

**Fig. 8:**
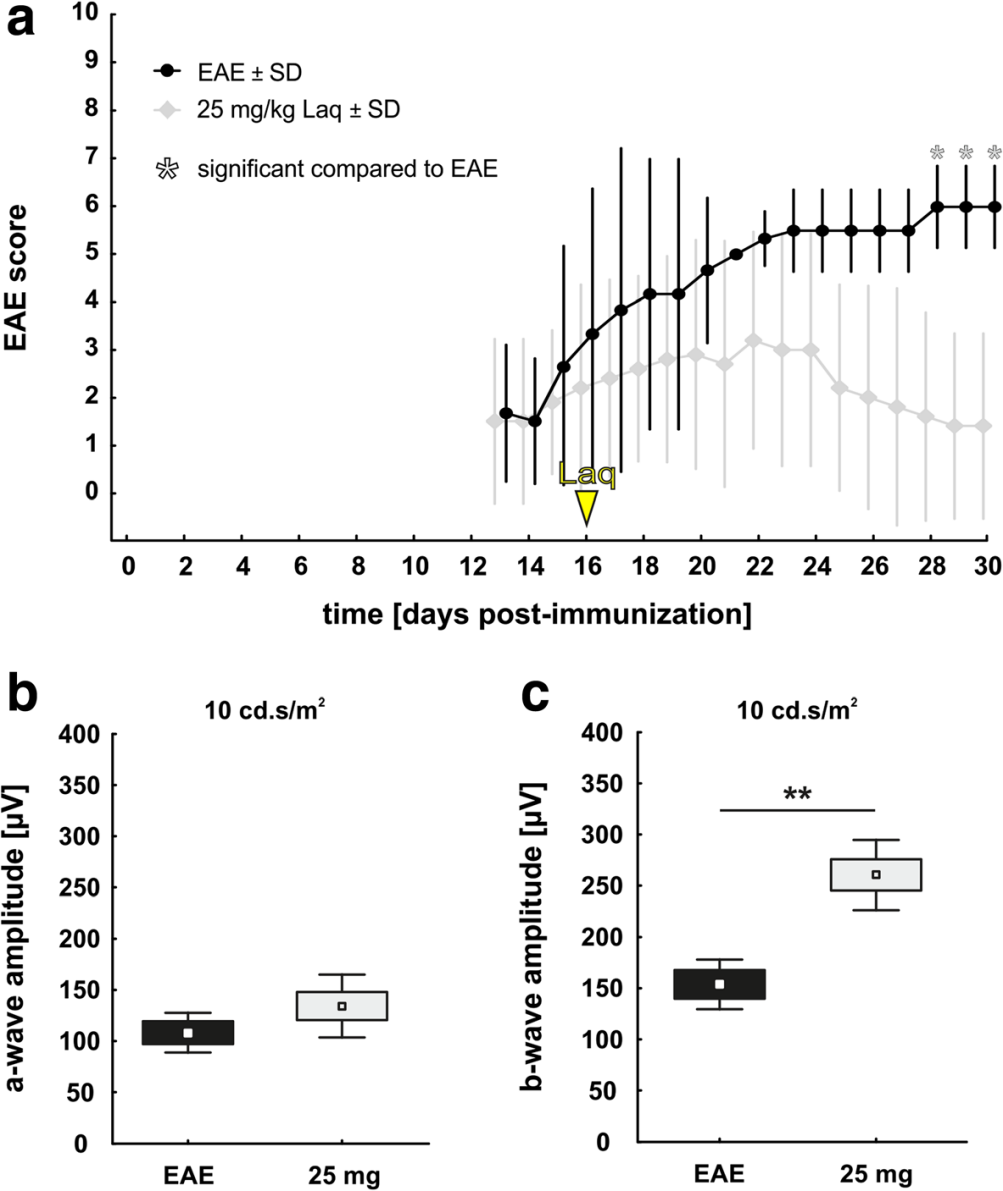
Clinical effects of therapeutic laquinimod treatment. **a** Mean clinical EAE scores after immunization with MOG_35–55_ peptide. Yellow arrowhead: start of laquinimod treatment. **b** Electroretinograms were measured at day 30. A-wave amplitudes illustrate conductivity of photoreceptors. **c** B-wave amplitudes illustrate conductivity of the inner nuclear layer. Values represent mean ± SD in **a**, mean ± SD ± SEM in **b** and **c**. Mann-Whitney *U* test for **a**; Student’s *t* test for **b** and **c**. *N* = 3–5/group in **a**–**c**. *p < 0.05, **p < 0.01

To investigate the effect of a delayed therapy, EAE animals received laquinimod (25 mg/kg) from day 16 on, since 60% of the animals had developed clinical signs by then. Laquinimod treatment led to a decreased EAE score (Fig. 8a). From day 28 to 30, the EAE score of the treated group was significantly lower than in the EAE group (*p* = 0.04).

ERG measurements, at the flash intensity of 10 cd.s/m^2^, revealed no changes in the a-wave amplitude between EAE and laquinimod-treated animals (*p* = 0.2) (Fig. 8b). However, the 25 mg/kg laquinimod group displayed a significantly improved b-wave amplitude compared to the EAE group at a flash intensity of 10 cd s/m^2^ (*p* = 0.003) (Fig. 8c).

**Fig. 9:**
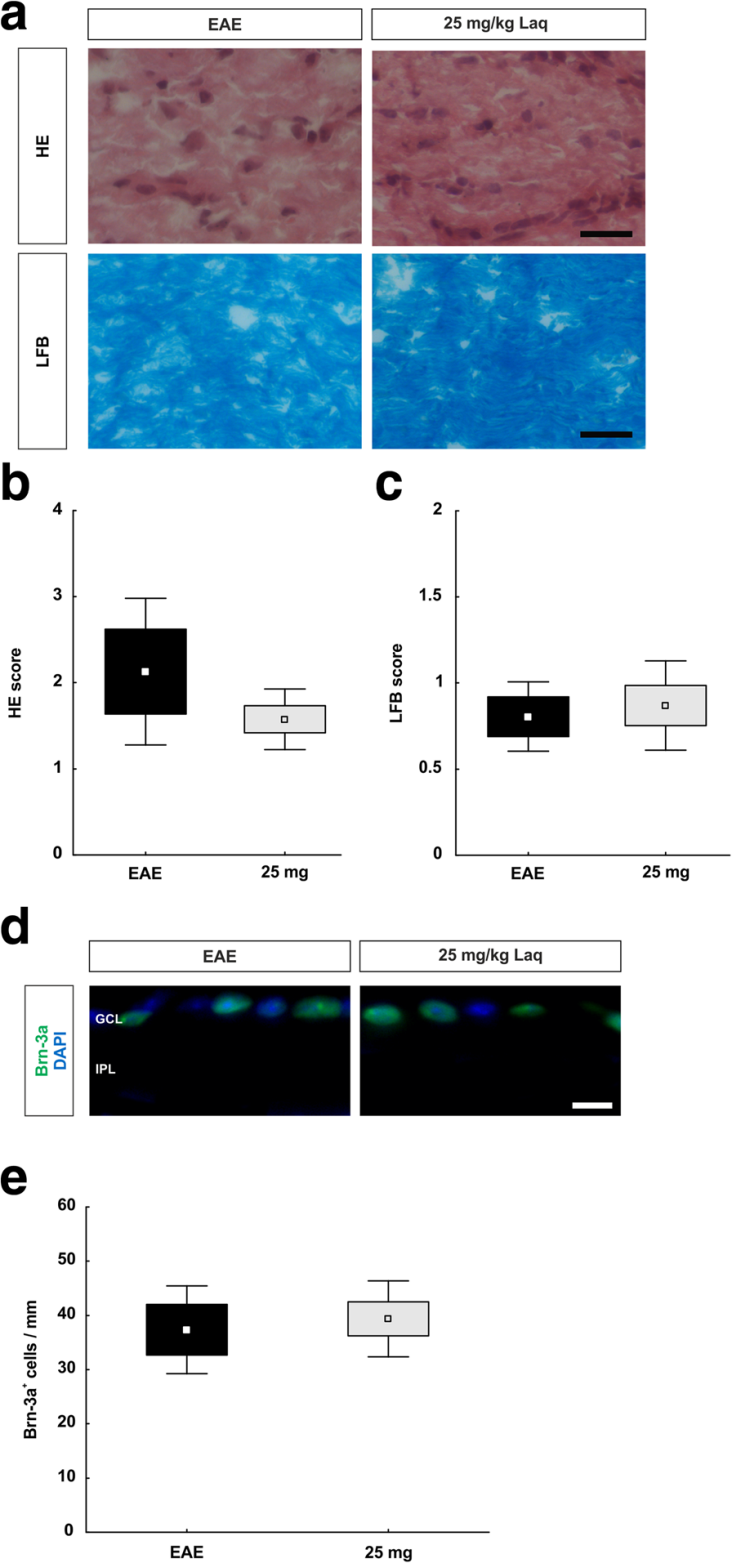
Therapeutic laquinimod treatment had no effect on optic nerve structure and retinal ganglion cells. **a** Optic nerves of the EAE and 25 mg/kg group were stained with HE and LFB. **b** Cellular infiltration was measured via HE score. **c** Demyelination was analyzed via LFB score. **d** Retinal ganglion cells were marked with Brn-3a antibody (green). **e** Numbers of retinal ganglion cells/mm. Values represent mean ± SD ± SEM in **b**, **c**, and **e**. Mann-Whitney *U* test for **b** and **c**; Student’s *t* test for **e**. *N* = 3–5/group in **a**–**c**. Scale bars: 20 μm in **a**; 10 μm in **c**. GCL = ganglion cell layer, IPL = inner plexiform layer

To evaluate the effects of a delayed therapy on optic nerves, sections were stained with HE and LFB (Fig. 9a) and scored. Regarding the HE score, no distinct differences were observed between the treatment group (1.58 ± 0.16) and the EAE animals (2.13 ± 0.49; *p* = 0.55) (Fig. 9b). Additionally, LFB staining did not reveal any differences between optic nerves of treated mice and those of the EAE group (25 mg/kg: 0.87 ± 0.12; EAE: 0.81 ± 0.12; *p* = 1.0) (Fig. 9c). Demyelination was comparable in both groups.

Retinal ganglion cells were labeled with a Brn-3a antibody (Fig. 9d). We observed no alteration in the number of Brn-3a^+^ cells in treated animals (39.39 ± 3.14 cells/mm) in comparison to retinas of the EAE group (37.37 ± 4.68 cells/mm; *p* = 0.7) (Fig. 9e). Loss of retinal ganglion cells occurred equally in both groups.

## Discussion

We investigated potential effects of laquinimod on the visual system in MOG_35–55_-immunized EAE mice. We found that laquinimod treatment protected optic nerves from EAE typical immune cell infiltration and demyelination. It reduced microglia and macrophages in both retina and optic nerve and decreased retinal macroglia. Moreover, it rescued retinal ganglion cells from apoptosis and conserved the electrical output of the inner nuclear layer. Delayed treatment improved clinical signs and partly retinal function.

### Reduced autoimmune response in optic nerve and retina

In EAE, microglial activation, phagocyte infiltration, and T-cellular influx are assumed to generate inflammatory lesions which are linked with demyelination and axonal injury in the spinal cords, brains [13], and optic nerves [17] of immunized animals.

Measurements in human MS lesions revealed six to 12 times higher numbers of macrophages than T cells [39]. Additionally, in the course of EAE microglia and macrophage levels remain higher than T-lymphocyte levels, the latter decreasing after an initial peak [40]. These findings indicate a major role of microglia and macrophages in MS. Consequently, the focus of our work lies on microglia and recruited phagocytes, as T-cell responses have already been monitored under laquinimod therapy in several studies [41–44].

Microglia have adverse functions: in a regular balance, they monitor the CNS [45, 46] and remove cellular detritus [47], whereas in EAE they fuel inflammation and neurotoxic processes [17, 48–51]. MS patients without activated microglia in their lesions show better outcomes of disease [52, 53]. In our study, we differentiated between resting microglia and infiltrating macrophages [35, 54, 55]. Administration of laquinimod led to reduced numbers of microglia in both optic nerve and retina. This correlates with findings by Mishra et al. in spinal cords of EAE mice, where laquinimod diminished numbers and activation of microglia [56]. The authors discuss the underlying mode of action to be a preservation of miR124a and interference with several signaling pathways for microglial activation. Yet, active microglia were increased in EAE, but not reduced under therapy in our findings.

Recruited phagocytes formed a remarkably larger proportion than microglia in our model, in which we explanted optic nerves and eyes in the beginning chronic phase of EAE. This matches observations that in the course of lesion development in MS, a shift from resident microglia to recruited, blood-derived macrophages takes place [55]. Laquinimod diminished numbers of recruited phagocytes.

### Preservation of optic nerve structures

Tissue infiltration with inflammatory cells causing demyelination was observed in the optic nerve in several EAE studies before [14, 15, 29]. In our study, we detected a reduced degree of cellular infiltration, especially recruited phagocytes, under laquinimod therapy.

Nevertheless, also T cells can be part of the inflammatory infiltrates in the context of autoimmune disorders [13, 16]. Laquinimod regulates T-cellular cytokine levels in favor of the anti-inflammatory T_H_2 and T_H_3 subtype [43] and downregulates pro-inflammatory T_H_1 cytokines, especially interferon (IFN)-γ and tumor necrosis factor (TNF)-α [42].

The infiltration with inflammatory cells is linked with demyelination in EAE optic nerves [15]. A protective effect of laquinimod on myelin sheaths was observed in our assessment. A negative correlation between axonal integrity and the number of microglia/macrophages can be found [56]. Thöne et al. suggest an upregulation of brain-derived neurotrophic factor to be the underlying myelin-protective mode of action in laquinimod [57]. Generally, the extent of demyelination to a certain degree depends on the type of EAE model that is applied: MOG-induced EAE lesions in mice display a high degree of global tissue injury, whereas the extent of primary demyelination is far lower [13]. Moreover, particularly, in C57BL/6 mice, MOG_35–55_ immunization does not cover auto-antibody-triggered destruction of myelin sheaths [13]. Therefore, our results on cellular infiltration in the optic nerve might be more conclusive than the degree of demyelination we measured. A delayed onset of treatment did not preserve the structure of the optic nerves. We assume that the inflammation-induced damage was already too severe. Our findings concerning the impact of laquinimod on optic nerve structures are congruent with investigations on mice brains in EAE, in which laquinimod was already shown to reduce both inflammatory infiltration and demyelination [58, 59].

### Reduced retinal macroglia response

Regarding macroglia, we focused on astrocytes. In CNS injuries, astrocytes can leave their normal state and become reactive forming a glial scar [60–62]. Thus, astrocytes can display dichotomic effects as reviewed for MS by Correale et al.: recruitment of immune cells, secretion of cytotoxic factors and inhibition of remyelination and axonal regeneration are opposed to modulation of the blood brain barrier integrity, improved viability of neurons, and induction of remyelination [61]. In a chronic MOG_35–55_ EAE model in C57/BL6 mice, the point in time of disease was shown to be of vital importance for the role of astrocytes: in the acute phase of EAE (days 0 to 15 post-immunization), depletion of reactive astrocytes aggravated clinical symptoms, whereas in the chronic phase (days 30 to 50) absence of reactive astrocytes reduced EAE scores [63]. From this, a negative impact of reactive gliosis in late EAE can be deduced. Performing explants in the beginning chronic phase, we observed that administration of laquinimod decreased retinal macroglia signals and therefore seemed to reduce EAE-induced reactive gliosis in the retina, but not in the optic nerve. Earlier experiments have shown difficulties in the quantification of macroglia signal in the optic nerve, as strong structural degeneration in EAE impedes area analyses [17].

In cuprizone-induced CNS demyelination, the effect of laquinimod on astrocytes was reported as a reduction of NF-κB activation in astrocytes and a diminished production of astrocytic pro-inflammatory cytokines [64]. The decreasing counts of retinal microglia we observed may also be a direct effect of astrocyte reduction, as less microglial infiltration was found in the retinas of GFAP and vimentin knockout mice [65].

### Protection of retinal ganglion cells and preserved conductivity of the inner nuclear layer

Retinal ganglion cell loss is common in EAE [15, 17, 47, 66]. The underlying mechanism is bilaterally discussed: most studies postulate that retinal ganglion cell loss represents a secondary effect of optic nerve inflammation [67–69], whereas current findings from human optic coherence tomography propose a development independent of optic nerve pathologies [70]. A breakdown of the blood retina barrier might be decisive here [66], as this provides migration of inflammatory cells, such as macrophages, into the retina. In our study, administration of laquinimod was linked with preserved retinal ganglion cell numbers. This could be due to diminished retinal microglia activation and macrophage infiltration as well as reduced astrocytic NF-κB activation and reduced apoptotic mechanisms under laquinimod treatment. In line with that, inhibition of NF-κB is associated with stagnation of retinal ganglion cell death in EAE mice [71]. However, a delayed treatment did not have any effects on the number of retinal ganglion cells in the 25 mg/kg laquinimod group. Since the structure of the optic nerves could also not be preserved, it is likely that inflammation-induced damage was already irreversible.

Next to retinal ganglion cell death, neuronal degeneration of the inner nuclear layer forms another retinal symptom in MS [72]. Accordingly, in our scotopic ERG measurements, we detected a diminished electrical output of neurons of the inner nuclear layer (INL) in EAE mice. With regard to their electrical output, neurons of the inner nuclear layer seem to be susceptible to retinal pathologies, as ERG measurements in another model of retinal degeneration, the ischemia-reperfusion model, already revealed [28]. Laquinimod nearly preserved the electrical output of the INL, which forms a retinal equivalent to the reduction of axonal optic nerve injury that could be shown. When treatment was started later, some preservation of retinal function could be observed.

### Influence of dosage

Several studies illustrate that laquinimod influences EAE disease severity in a dose-dependent manner [21, 43, 73]. Congruent with the results from the Brueck group, we found the strongest effect for treatment with 25 mg/kg and smaller effects with 5 and 1 mg/kg. Despite its beneficial influence on EAE scores, in our further investigations, the 1 mg/kg dose had no detectable effect on markers of inflammation and neurodegeneration in the optic nerve or retina. Laquinimod is known to pass the blood brain barrier, regardless of its integrity. Yet, in EAE mice, exposure in the CNS measures only 13% of the peripheral blood concentration [73], which explains the need of relatively high doses. Remarkably, 5 mg/kg laquinimod achieved better results concerning the decrease of retinal ganglion cell apoptosis rates and conductivity of retinal INL than 25 mg/kg did. Whether this suggests bivalent, maybe cytotoxic effects of the highest dose on retinal ganglion cells and neurons of the INL requires further investigation.

## Conclusion

The novel oral immunomodulatory agent laquinimod is known to exert neuroprotective and anti-inflammatory effects on the spinal cord and brain. Our study delivered evidence that these findings are transferable to the optic nerve and retina, which are affected first in MS. Doses of 5 and 25 mg/kg laquinimod attenuated MS-related pathologies in the optic nerve and retina in an EAE model. Later onset of treatment also led to some improvement of clinical signs and retinal function, but could not prevent demyelination and retinal ganglion cell loss. We were able to corroborate the positive effect of laquinimod on neurological impairment in EAE, when treatment was started early enough. As the agent showed a positive impact on the visual system, which forms a crucial spot of manifestation in MS, its neuro-ophthalmic effect might be an interesting subject of further investigations in the future.

## Additional file

Additional file 1: Analyses of different mRNAs via qRT-PCR. Values are median and interquartile range. Significant differences are marked in bold. (DOCX 16.4 kb)

## Abbreviations

AMWAP: Activated microglia/macrophage whey acidic protein
CNS: Central nervous system
CO: Control group
Ct: Threshold cycle
DAPI: 4′,6-Diamidino-2-phenylindole
EAE: Experimental autoimmune encephalomyelitis
ERG: Electroretinogram
GCL: Ganglion cell layer
GFAP: Glial fibrillary acidic protein
HE: Hematoxylin and eosin
HSD: Honest significant difference
Iba1: Ionized calcium-binding adapter molecule 1
IFN-β: Interferon β
IFN-γ: Interferon γ
INL: Inner nuclear layer
IPL: Inner plexiform layer
IQR: Interquartile range
LFB: Luxol fast blue
MBP: Myelin basic protein
miR124a: MicroRNA124a
MOG: Myelin oligodendrocyte glycoprotein
MRI: Magnetic resonance imaging
MS: Multiple sclerosis
NFL: Nerve fiber layer
NF-κB: Nuclear factor kappa-light-chain-enhancer of activated B cells
PBS: Phosphate-buffered saline
qRT-PCR: Quantitative real-time polymerase chain reaction
SD: Standard deviation
T_H_1: T helper cell type 1
T_H_2: T helper cell type 2
T_H_3: T helper cell type 3
Tmem119: Transmembrane protein 119
TNF-α: Tumor necrosis factor α
