# Supplementary material for: Laquinimod protects the optic nerve and retina in an experimental autoimmune encephalomyelitis model
Source: J Neuroinflammation. 2018 Jun 14;15:183. doi: 10.1186/s12974-018-1208-3 (PMC6002998; doi:10.1186/s12974-018-1208-3)
Supplement: Supplementary file 1 — Analyses of different mRNAs via qRT-PCR. Values are median and interquartile range. Significant differences are marked in bold. (DOCX 16.4 kb) [file 12974_2018_1208_MOESM1_ESM.docx]

**Additional file 1** Analyses of different mRNAs via qRT-PCR. Values are median and interquartile range. Significant differences are marked in bold.

|  | **compared to CO** | | | **compared to EAE** | |
| --- | --- | --- | --- | --- | --- |
|  | **EAE** | **5 mg/kg Laq** | **25 mg/kg Laq** | **5 mg/kg Laq** | **25 mg/kg Laq** |
| **β-actin** | 1.155 | 1.155 | 1.155 | 0.98 | 0.97 |
| **Cyclophilin** | 0.865 | 0.865 | 0.865 | 1.019 | 1.032 |
| **Brn-3a** | 0.62 (0.37-0.95) | 0.78 (0.49-1.13) | 1.36 (1.00-1.96) | 1.26 (0.77-1.78) | 1.56 (0.98-2.50) |
| **p-value** | **0.036** | 0.28 | 0.092 | 0.33 | 0.074 |
| **Iba1** | 2.41 (1.78-3.47) | 2.42 (1.85-2.95) | 1.29 (0.87-2.36) | 1.00 (0.75-1.36) | 0.38 (0.25-0.56) |
| **p-value** | **<0.001** | **0.002** | 0.21 | 0.99 | **0.003** |
| **Tmem119** | 2.27 (1.60-3.55) | 2.11 (1.53-2.89) | 1.45 (1.00-2.17) | 0.87 (0.63-1.47) | 0.57 (0.38-0.91) |
| **p-value** | **0.006** | **0.01** | 0.15 | 0.5 | **0.038** |
| **AMWAP** | 2.27 (1.55-3.78) | 2.45 (1.50-4.25) | 1.40 (1.04-1.93) | 1.06 (0.69-1.83) | 0.64 (0.37-1.01) |
| **p-value** | **0.024** | **0.017** | 0.07 | 0.8 | 0.06 |
| **CD68** | **3.01 (2.35-3.83)** | **2.41 (1.49-3.66)** | **1.85 (1.44-2.41)** | **0.80 (0.49-1.19)** | **0.53 (0.35-0.76)** |
| **p-value** | **0.002** | **0.002** | **0.003** | **0.3** | **0.008** |
| **GFAP** | 2.23 (1.05-5.02) | 3.13 (2.21-4.70) | 1.88 (1.34-2.81) | 1.40 (0.69-2.73) | 0.84 (0.42-1.64) |
| **p-value** | **0.03** | **0.003** | **0.006** | 0.4 | 0.6 |
